# Warfarin Failure in a Patient With Chronic Thromboembolic Pulmonary Hypertension: A Case Report and Literature Review

**DOI:** 10.7759/cureus.27007

**Published:** 2022-07-19

**Authors:** Tsering Dolkar, Aysham Chaudry, Ferdous Salauddin, Nway Nway, Nevil Kadakia, Madhumati Kalavar, Muhammad H Dogar

**Affiliations:** 1 Internal Medicine, One Brooklyn Health (OBH) Interfaith Medical Center, Brooklyn, USA; 2 Medicine, Touro College of Osteopathic Medicine, Middletown, USA; 3 Hematology and Oncology, One Brooklyn Health (OBH) Interfaith Medical Center, Brooklyn, USA; 4 Internal Medicine/Cardiology, One Brooklyn Health (OBH) Interfaith Medical Center, Brooklyn, USA

**Keywords:** supratherapeutic inr, deep vein thrombosis, massive pulmonary embolism, venous thromboembolism, chronic thromboembolic pulmonary hypertension, warfarin failure

## Abstract

Chronic thromboembolic pulmonary hypertension (CTEPH) is a form of pulmonary hypertension caused by chronic venous thromboembolism (VTE). Venous thromboembolism (VTE) manifests as deep vein thrombosis (DVT), progressing to pulmonary embolism (PE). Pulmonary endarterectomy (PEA) is the preferred therapeutic option as it provides vascular disobliteration. Long-term anticoagulation with warfarin or direct oral anticoagulants (DOACs) is recommended for patients at risk for recurrent DVT in poor surgical candidates. However, treatment failure remains a concern. We present a patient who had VTE despite long-term anticoagulation with warfarin who had failed treatment and developed VTE with a therapeutic dilemma to continue anticoagulation despite supratherapeutic international normalized ratio (INR).

## Introduction

Deep vein thrombosis (DVT) is the development of a blood clot within a deep vein, usually of a lower extremity. The Virchow triad is the main pathophysiological component observed in patients with VTE. It is characterized by hypercoagulability, endothelial damage, and venous stasis. The risk factors for the condition include endothelial damage possibly from trauma, venous stasis due to immobility, and hypercoagulability, which may be due to thrombophilia or thrombocytosis. These risk factors are collectively referred to as the Virchow triad [1]. A serious complication of DVT is the manifestation of pulmonary embolism, with the embolization of the thrombosis to the lungs through the inferior vena cava (IVC) causing luminal obstruction of one or more pulmonary arteries. The manifestation of DVT and pulmonary embolism is referred to as venous thromboembolism (VTE). Venous thromboembolism is the third leading cause of cardiovascular disease in the United States [2]. Chronic thromboembolic pulmonary hypertension (CTEPH) is a progressive form of pulmonary hypertension caused by chronic pulmonary thromboembolism.

## Case presentation

The patient is an 84-year-old female with a past medical history of chronic obstructive pulmonary disease (COPD), type 2 diabetes mellitus, hypertension, hyperlipidemia, heart failure, chronic peripheral vascular disease with bilateral above-the-knee amputation, chronic kidney disease stage 3b, essential thrombocytosis, and a history of recurrent DVTs with inferior vena cava (IVC) filter placed 10 years ago and on warfarin 2 mg daily for about five years. The patient had no history of recent trauma, injury, or surgery and did not have a prior history of cancer. The patient presented to the emergency department (ED) from a nursing home with shortness of breath for three days.

On physical examination, the patient was ill-appearing, lethargic, and dyspneic. Vital signs revealed an oxygen saturation of 82% on room air, respiratory rate of 22/minute, and heart rate of 75/minute. The patient’s cardiac examination was remarkable for a regular S1 and a prominent S2 without splitting. Respiratory examination showed coarse breath sounds with crackles in the upper lobes, more prominent on the right side. The patient’s musculoskeletal examination was remarkable for above-the-knee amputation of bilateral lower extremities with edema.

Laboratory results on admission showed elevated D-dimer, thrombocytosis, leukocytosis, hyperkalemia, hyperphosphatemia, and acute renal failure (Table 1).

**Table 1:** Laboratory findings on admission

Laboratory test	Normal range	Results
WBC	4.5-11 × 10^3^/uL	13.8
Hemoglobin	11-15 g/dL	10.5
Hematocrit	35%-46%	33.6
MCV	80-100 fL	112
Platelets	130-400 × 10^3^/uL	550
BUN	9.8-20.1 mg/dL	88.8
Creatinine	0.57-1.11 mg/dL	3.16
eGFR	≥90 mL/minute/1.73 m^2^	14
Potassium	3.5-5.1 mmol/L	5.2
Phosphorus	2.3-4.7 mg/dL	6.4
D-dimer	≤500 ng/mL DDU	3,355

The patient’s arterial blood gas showed hypoxemia (Table 2).

**Table 2:** Arterial blood gas on admission

Component	Normal range	Results
pH (arterial)	7.35-7.45	7.29
pCO2 (arterial)	35-45 mmHg	39.2
pO2 (arterial)	80-100 mmHg	46
HCO3 (arterial)	22-28 mmol/L	18.2
O2 saturation (arterial)	92%-98.5%	75.7

On admission, the international normalized ratio (INR) was found to be supratherapeutic (5.64), in view of which warfarin was stopped and the coagulation profile trended (Table 3).

**Table 3:** Coagulation profile trend since admission PT: prothrombin time, INR: international normalized ratio, PTT: partial thromboplastin time

Day	PT (9.8-13.4 seconds)	INR (0.85-1.15)	PTT (24.9-35.9 seconds)
Admission	69.5	5.64	43.5 (warfarin held)
1	79.3	6.38	46
2	111.4	9	55
3	86.7	6.97	56.3
4	61.7	4.98	50.1
5	29.6	2.42	77.1 (heparin started)
6	24.7	2.01	34
7	40.7	3.32	105 (heparin held)
8	76.1	6.12	78.9

INR remain elevated for few more days likely due to renal failure as warfarin is primary excreted via the kidneys. Factor V Leiden assay, JAK-2 genetic testing, antithrombin-3 activity, and lupus anticoagulant were negative. The patient had a previous bone marrow biopsy done, which revealed megakaryocytosis compatible with essential thrombocytosis.

**Figure 1:**
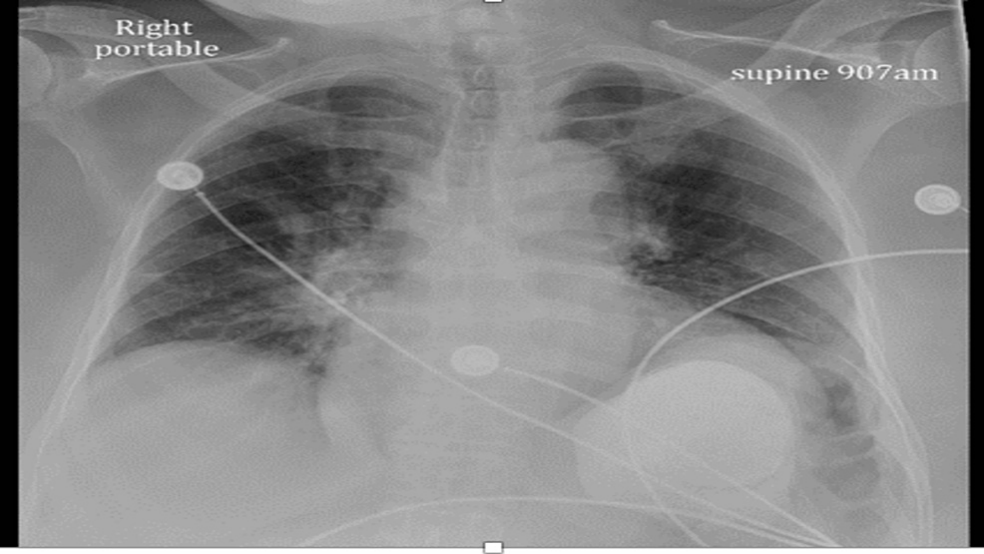
Chest X-ray showing a patchy left upper lung pulmonary opacity and patchy bibasilar subsegmental atelectasis

The patient developed shortness of breath three days after admission and started desaturating at 80% on room air. The patient was started on a non-rebreather mask for hypoxic respiratory failure. This patient had a high probability of pulmonary embolism. Chest X-ray showed a patchy left upper lung pulmonary opacity and patchy bibasilar subsegmental atelectasis (Figure 1).

**Figure 2:**
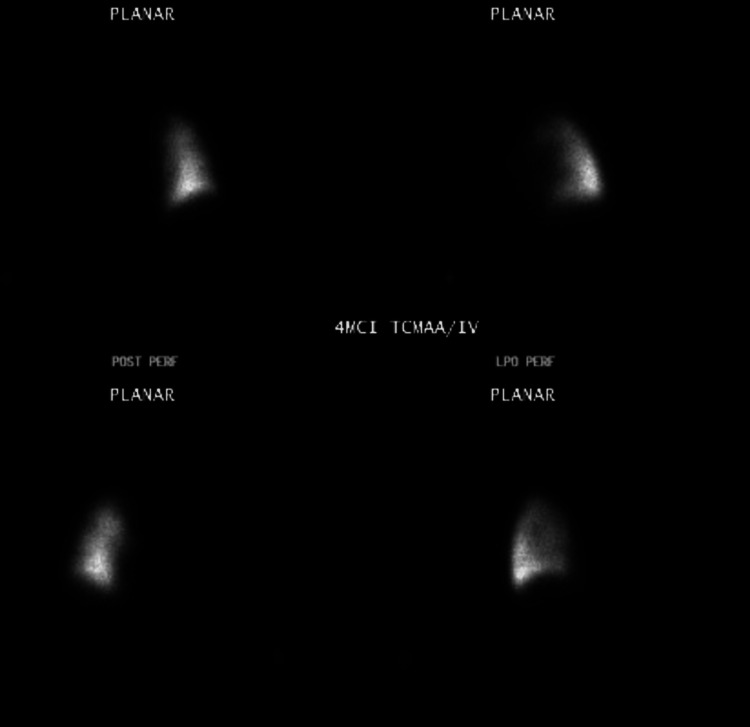
VQ scan showing the right lung with no left lung activity, compatible with embolic occlusion of the left main pulmonary artery

Ventilation-perfusion (V/Q) scan showed no left lung activity, compatible with embolic occlusion of the left pulmonary artery (Figure 2).

**Figure 3:**
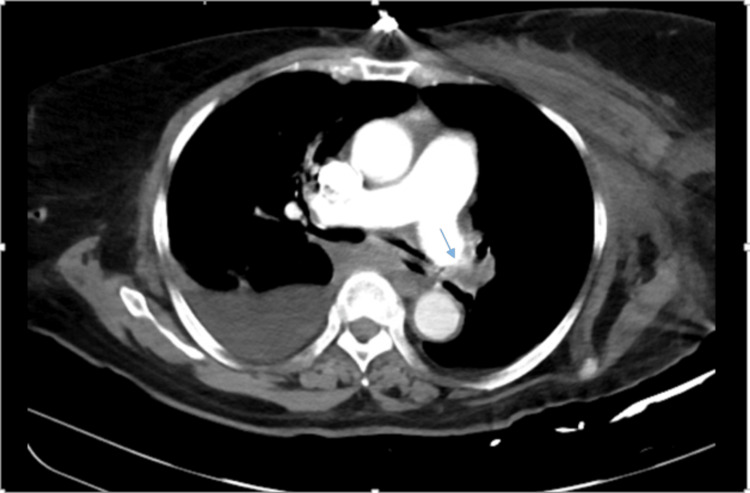
Chest CTPA with contrast showing large occlusion at the left main pulmonary artery (arrow) and right lung pleural effusion

A CT pulmonary angiogram (CTPA) of the chest with contrast showed a right-sided pleural effusion, diffuse infiltrates of the right lung, patchy infiltrates of the left lung, no peripheral blood vessels in the left lung, and occlusion of the left pulmonary artery (Figure 3). Transthoracic echocardiogram showed an ejection fraction of 55%-60% and a dilated and hypokinetic right ventricle with pulmonary arterial systolic pressure (PASP) of 90-100 mmHg. The patient met the diagnostic criteria for chronic thromboembolic pulmonary hypertension based on this data.

The patient was upgraded to the ICU and started on a heparin drip when the INR reached 2-3; soon after, PTT was 105 seconds, and heparin was held. The patient was a poor candidate for the open-heart surgery for embolectomy and a catheter-based invasive procedure due to age, functional capacity, and chronicity of the clot. The patient was on warfarin for CTEPH. Even after being compliant with warfarin and a supratherapeutic admission INR, the patient developed acute hypoxic respiratory failure secondary to pulmonary embolism and subsequently expired after 15 days of hospitalization.

## Discussion

Many patients with venous thromboembolism may develop chronic thromboembolic pulmonary hypertension (CTEPH). CTEPH occurs due to an incomplete resolution of VTE from pulmonary artery obstruction [3,4]. Certain hematological disorders, such as thrombocytosis, and autoimmune disorders are risk factors for the development of CTEPH [5]. The risk factors for VTE include a history of prolonged immobilization or hospitalization, peripheral vascular disease, obesity, previous VTE, recent surgery or trauma, malignancy, stroke with hemiplegia or immobility, age > 65 years, thrombocytosis, and heart failure [6,7]. More recently, COVID-19 infection within three months has been associated with VTE [8]. Our patient had multiple risk factors, including a history of recurrent DVT with bilateral above-the-knee amputation, older age, obesity, essential thrombocytosis, heart failure, and a recent infection with COVID-19.

Warfarin inhibits the gamma-carboxylation of factors II, VII, IX, and X, thereby preventing their function in coagulation [9]. Warfarin use requires constant monitoring of patient INR. Several factors such as heart failure, acute illnesses, and medication interactions may lead to a supratherapeutic INR. A subtherapeutic INR would require adjustment of the dose of warfarin. Our patient had a supratherapeutic INR on regular monitoring yet developed warfarin failure, in contrast to warfarin inefficiency where therapeutic INR is not reached.

Although a rare occurrence, warfarin failure has been observed in a few other cases. In these cases, despite a therapeutic INR and a high suspicion of malignancy, there was a recurrence of venous thromboembolism in a patient on warfarin [10]. More recently, warfarin failure has been observed in patients due to a hypercoagulable state after infection with COVID-19 despite the placement of an IVC filter [11]. One study concluded that patients with recurrent VTE have an increased incidence of recurrence despite being on warfarin therapy [12]. Our patient’s hypercoagulable state may be attributed to CTEPH or a COVID-19 infection two months before the onset of her recurred VTE.

The initial management of VTE and CTEPH is anticoagulation [13]. After the initial management of VTE, the patient is switched to long-term anticoagulation, either through oral anticoagulants or parenteral subcutaneous anticoagulants. Among the oral anticoagulants, direct oral anticoagulants (DOAC) are preferred in nonpregnant patients without renal insufficiency. In patients with renal insufficiency, warfarin is the preferred anticoagulant. Low-molecular-weight heparin (LMWH) is generally used in patients with malabsorption who cannot tolerate oral medications. If LMWH is contraindicated due to heparin-induced thrombocytopenia, the patient is placed on fondaparinux. Our patient had long-term anticoagulation through warfarin due to renal insufficiency. Warfarin resistance presents with a subtherapeutic INR and can be seen in some patients due to a multitude of reasons such as poor absorption, high vitamin K intake, and rapid drug deactivation. In contrast, warfarin failure presents with a supratherapeutic INR with recurrent thrombosis, as seen in this patient and also reported earlier [11,12]. Cases with treatment failure may warrant reevaluation of the current guideline of treatment.

Pulmonary endarterectomy (PEA) is the preferred treatment for CTEPH as it provides a potential curable option, and if the patient is not a good surgical candidate like our patient, oral anticoagulation is the alternative choice. Patients with CTEPH should receive lifelong anticoagulation, and pulmonary endarterectomy (PEA) is the only potentially curable treatment option. The decision on how to treat patients with CTEPH should be made at an expert center based upon interdisciplinary discussion among internists, radiologists, and expert surgeons [14].

Newer medication such as riociguat, which is a first-in-class soluble guanylate cyclase stimulator, approved for the treatment of adults with pulmonary arterial hypertension (PAH), inoperable chronic thromboembolic pulmonary hypertension (CTEPH), or persistent or recurrent CTEPH after pulmonary endarterectomy has been shown to improve exercise tolerance [15]. Our patient was bedridden with bilateral above-the-knee amputation, and it would not be possible to assess the efficacy of riociguat in this patient, so it was not offered to her.

For our patient, we were faced with a therapeutic dilemma whether to treat despite bleeding risk as the patient had supratherapeutic INR with gastrointestinal bleeding and the progression of respiratory failure due to CTEPH. Another challenge for the management is that due to the multitude of comorbidities, she was not a candidate for any surgery or invasive procedures such as right heart catheterization. We employed the comprehensive approach by multidisciplinary team efforts including surgery, pulmonary, hemato-oncology, and cardiology.

## Conclusions

CTEPH is one of the leading causes of severe pulmonary hypertension. Lifelong anticoagulation is the mainstay of therapy for patients who are not good surgical candidates. An anticoagulation failure such as warfarin like our patient can lead to a series of challenges in the management and also the progression of the CTEPH. This case may increase awareness of the therapeutic warfarin failure in the treatment of CTEPH for future guidelines to balance the risk of bleeding and hypercoagulation.
